# A Retrospective Cohort Study Evaluating the Safety and Efficacy of Sequential versus Concurrent Intrapleural Instillation of Tissue Plasminogen Activator and DNase for Pleural Infection

**DOI:** 10.1155/2023/6340851

**Published:** 2023-12-18

**Authors:** Ken Junyang Goh, Wui Mei Chew, Jasmine Chiat Ling Ong, Carrie Kah-Lai Leong, Imran Bin Mohamed Noor, Devanand Anantham, Li Yan Sandra Hui, Mindy Chu Ming Choong, Charlene Jin Yee Liew, Marnie Tamayo Gutierrez, Jane Jing Yi Wong, Ivana Gilcrist Chiew Sian Phua, Wen Ting Lim, Qiao Li Tan

**Affiliations:** ^1^Department of Respiratory and Critical Care Medicine, Singapore General Hospital, Singapore; ^2^Department of Respiratory and Critical Care Medicine, Changi General Hospital, Singapore; ^3^Division of Pharmacy, Singapore General Hospital, Singapore; ^4^Department of Diagnostic Radiology, Singapore General Hospital, Singapore; ^5^Department of Diagnostic Radiology, Changi General Hospital, Singapore; ^6^Advanced Practice Nurse Development, Changi General Hospital, Singapore; ^7^Division of Nursing, Singapore General Hospital, Singapore

## Abstract

**Methods:**

We conducted a retrospective review of patients with pleural infection requiring intrapleural therapy at two tertiary referral centres.

**Results:**

We included 84 (62.2%) and 51 (37.8%) patients who received sequential and concurrent intrapleural therapy, respectively. Patient demographics and clinical characteristics, including age, RAPID score, and percentage of pleural opacity on radiographs before intrapleural therapy, were similar in both groups. Treatment failure rates (defined by either in-hospital mortality, surgical intervention, or 30-day readmission for pleural infection) were 9.5% and 5.9% with sequential and concurrent intrapleural therapy, respectively (*p* = 0.534). This translates to a treatment success rate of 90.5% and 94.1% for sequential and concurrent intrapleural therapy, respectively. There was no significant difference in the decrease in percentage of pleural effusion size on chest radiographs (15.1% [IQR 6-35.7] versus 26.6% [IQR 9.9-38.7], *p* = 0.143) between sequential and concurrent therapy, respectively. There were also no significant differences in the rate of pleural bleeding (4.8% versus 9.8%, *p* = 0.298) and chest pain (13.1% versus 9.8%, *p* = 0.566) between sequential and concurrent therapy, respectively.

**Conclusion:**

Our study adds to the growing literature on the safety and efficacy of concurrent intrapleural therapy in pleural infection.

## 1. Introduction

The incidence of pleural infection continues to rise and remains associated with significant mortality [1, 2] and morbidity. Over 80,000 new cases of pleural infections are diagnosed in the United Kingdom and United States each year, with an overall mortality of 10-20% [3]. First-line therapy consisting of antibiotics and pleural drainage fails in about 30% of patients [4], and surgery for fluid drainage and decortication presents higher risks for patients with significant comorbidities or frailty. Intrapleural therapy with tissue plasminogen activator (tPA) and deoxyribonuclease (DNase) has been established as an alternative to surgery, with the multicentre intrapleural sepsis trial (MIST-2) and subsequent studies demonstrating promising treatment success rates [5, 6], as defined by radiographic clearance and avoiding surgical intervention for pleural infection. Recent meta-analyses have further demonstrated the efficacy of intrapleural therapy, with significant reduction in the need for surgical intervention and overall treatment failure [7, 8].

The MIST-2 protocol involves sequential instillations of tPA and DNase. Intrapleural tPA instillation is followed by clamping of the chest drain for one hour, unclamping for drainage for another hour, and then repeating the same process for DNase. The chest drainage system is accessed up to twelve times for six doses, potentially increasing the risk of drain dislodgement and risk of infection. This process also increases the workload for healthcare workers, particularly when some doses are administered after office hours. This may also affect compliance to the protocol, as observed by a compliance rate of about 70% in the MIST-2 trial [5], which may be further reduced in real-world settings.

An alternative method is the concurrent instillation of intrapleural therapy, which reduces the complexity of the administration process [9]. The use of concurrent therapy decreases the need for healthcare workers to access the chest drain, shortens the treatment time from 3 hours to 2 hours, and is easier to implement, particularly after office hours. These are likely to lead to significant reduction in workload and increased compliance to therapy, which are important aspects of treatment that remain under-recognised in the treatment of pleural infections.

A few centres have published small cohort studies reporting similar outcomes and safety comparable to sequential instillation of tPA/DNase [10–14]. Interestingly, a recent survey of respiratory physicians showed that most respondents (61%) adopt concurrent instillation of intrapleural therapy in their institutions [15]. However, the evidence for concurrent intrapleural therapy, which is based on small studies or studies lacking a comparator arm, remains unclear [9]. In this study, we aim to compare the safety and efficacy of sequential versus concurrent intrapleural tPA/DNase in the treatment of pleural infection.

## 2. Methodology

We performed a retrospective review of consecutive patients who received intrapleural tPA/DNase for pleural infection from Singapore General Hospital (SGH) between January 2017 and September 2022 and Changi General Hospital (CGH) between January 2021 and June 2022. Administration of concurrent intrapleural therapy started in Sept 2021 and June 2021 in SGH and CGH, respectively. Patients were identified from a pleural database, as well as screening hospital administration codes for intrapleural alteplase. Pleural infection was defined as a clinical history compatible with pleural infection and one or more of the following pleural fluid characteristics: (i) purulent macroscopically, (ii) positive gram stain or culture, (iii) pH ≤ 7.20, glucose ≤ 3.0 mmol/L, or lactate dehydrogenase (LDH) > 1,000 IU/L, and (iv) complex pleural effusion defined by ultrasonographic or radiographic evidence of loculations or septations.

In both hospitals, tPA (alteplase) and DNase were separately reconstituted in 30-50 mL of 0.9% saline before intrapleural instillation. For sequential intrapleural therapy, tPA followed by a 10-20 mL saline flush was instilled, and the chest drain was clamped for one hour and then opened for drainage for another hour, before repeating the same procedure with DNase. For concurrent intrapleural therapy, DNase was instilled immediately after tPA followed with a 10-20 mL saline flush, and the chest drain was clamped for one hour and then opened for drainage. Modifications to the dose of alteplase (using 5 mg as a first dose or for all doses) and frequency of administration (once-daily dosing instead of twice daily) were determined by managing physicians, based on clinical and radiological response. In addition, the selection and duration of antibiotics, application of external suction through the chest drain, timing of treatment, and referral to surgery were left to the discretion of managing physicians.

Hospital medical records were reviewed for patient demographics, laboratory data, patient outcomes, and complications of intrapleural therapy. These were also used to calculate the Charlson comorbidity score (CCI) and RAPID [16] (renal profile, age, purulence of pleural fluid, infection source, and dietary factors) score. The RAPID score has been validated in prospective studies to predict mortality in pleural infections [2] and is useful as a surrogate measure of pleural infection severity.

The primary outcome was treatment failure as defined by either in-hospital mortality attributed to pleural infection, surgical intervention within 30 days following the initial dose of tPA/DNase, or readmission for pleural infection 30 days following hospital discharge from the index hospitalization. The secondary outcomes were the percentage change of the area of pleural opacity on chest radiographs and the adverse event rate. The percentage change in pleural opacity was determined by comparing chest radiographs after chest drain insertion (but before intrapleural therapy) with chest radiographs following intrapleural tPA/DNase. These were graded by two thoracic radiologists (one for each hospital) using previously published methods [5]. Both radiologists were blinded to the mode (sequential versus concurrent) of intrapleural therapy. Adverse events were pleural bleeding, defined as bloody pleural fluid associated with an acute drop in hemoglobin requiring blood transfusion or surgical intervention, and chest pain requiring escalation in analgesia.

Descriptive statistics of the variables were expressed with mean and standard deviation, median with interquartile range, or numbers with percentage. All statistical analyses were performed using SPSS statistics software version 22.0 (IBM Corp, Armonk, US). Comparisons between groups were made with the Wilcoxon signed-rank test and chi-square test, and a *p* value of < 0.05 was considered significant.

## 3. Results

### 3.1. Patient Demographics and Clinical Characteristics

A hundred and thirty-five patients (77.8% male; median age: 64 years) from SGH (*n* = 104) and CGH (*n* = 31) were included (Table 1). Eighty-four (62.2%) patients received sequential, and fifty-one (37.8%) patients received concurrent intrapleural therapy. The median RAPID score was 3 (interquartile range (IQR): 2-4), and 21.5% of patients had a high-risk RAPID score (≥5) (Table 2). Most patients (91.9%) had loculated pleural effusions or septations visualized on ultrasonography or CT (computed tomography) imaging. Pleural fluid biochemistry showed a median glucose of 3.0 mmol/L (IQR: 0.6-4.9) and LDH of 2225 IU/L (IQR: 982-4313). A positive pleural fluid gram stain or culture was present in 40.0% of patients, with *Streptococcus viridans* being the most common (44.2%) organism isolated (Table 3).

### 3.2. Treatment Characteristics and Outcomes

Patients were initiated on intrapleural therapy at a median of 2 days (IQR 1-4) after chest drain insertion (Table 4). The number of tPA/DNase doses was variable, with 29.6% of patients receiving 1 to 2 doses, 39.3% receiving 3 to 4 doses, and 31.1% of patients receiving 5 to 6 doses. Once-daily dosing of tPA/DNase was administered in 20.7% of patients, and alteplase dosing was deescalated (5 mg Alteplase given as the first dose or all doses) in 11.1% of patients.

Chest radiographs pre- and post-intrapleural therapy for comparison were available for 127 patients. Postintrapleural therapy chest radiographs were performed between 3 and 7 days following the first dose of intrapleural therapy. The estimated area of hemithorax occupied by pleural effusion decreased from a median of 35.3% (IQR: 16.5–51.9) to 10.0% (IQR: 5.0–16.5) (*p* < 0.001) following intrapleural therapy. Treatment failure occurred in 11 (8.1%) patients: two patients required surgery, six patients with mortality from pleural infection, and three patients were readmitted for persistent pleural infection, of whom one required surgery (Table 5). This translates to an overall treatment success rate of 91.8%. There were five deaths due to unrelated causes. Two patients died from active cancer (acute myeloid leukemia and metastatic esophageal cancer) and three patients died from hospital-acquired pneumonia that occurred after the resolution of the pleural infection: one patient developed aspiration pneumonia on the contralateral side from the pleural infection, one patient passed away from COVID-19 pneumonia, and the third patient passed away from hospital-acquired pneumonia approximately three months after resolution of the pleural infection.

Approximately one quarter (25.2%) of patients had failure of intrapleural therapy or an adverse event: 6.7% of patients had a pleural bleeding, and 11.9% had chest pain requiring escalation of analgesia. Among the nine patients with pleural bleeding, three had bleeding risk: one patient receiving concurrent intrapleural therapy was on treatment dose of low molecular weight heparin, one patient receiving concurrent intrapleural therapy was on aspirin with elevated urea (21.0 mmol/L), and one patient receiving sequential therapy had thrombocytopenia (44 × 10^9^/L) with elevated urea (28.9 mmol/L) at the time of interpleural therapy. Two patients required surgery for significant pleural bleeding. Pleural bleeding for the remaining seven patients resolved with conservative management, with all seven patients receiving one unit of blood transfusion each.

### 3.3. Comparison between Sequential and Concurrent Intrapleural tPA/DNase Therapy

There were no significant differences in age, CCI, percentage of pleural opacity on chest radiograph, RAPID score, or antiplatelet/anticoagulation therapy observed between the two patient groups (Table 5). A higher proportion of patients received the first dose of intrapleural therapy within 48 hours of chest drain insertion (70.6% versus 51.2%, *p* = 0.027) in the concurrent intrapleural therapy group. Patients who received concurrent intrapleural therapy also received less tPA/DNase doses (3 (IQR 2-3) versus 4 (IQR 3-6), *p* < 0.001) overall. More patients with concurrent intrapleural therapy (84.3%) received ≤3 doses, as compared to those with sequential intrapleural therapy (50%). However, a lower proportion of patients with concurrent intrapleural therapy had received a deescalated (5 mg alteplase) dose (3.9% versus 15.5%, *p* = 0.038).

There was no significant difference observed in treatment failure rates (9.5% versus 5.9%, *p* = 0.534) in patients receiving sequential and concurrent intrapleural therapy, respectively. This translates to a treatment success rate of 90.5% and 94.1% for sequential and concurrent intrapleural therapy, respectively. There was also no significant difference in the decrease in percentage of pleural effusion size on chest radiographs (15.1% (IQR:6.0-35.7) versus 26.6% (IQR: 9.9-38.7), *p* = 0.143), and overall rates of failed therapy and/or adverse events were similar (26.2% versus 25.5%, *p* = 0.928) between patients receiving sequential versus concurrent intrapleural therapy, respectively. There were no significant differences in pleural bleeding (4.8% versus 9.8%, *p* = 0.298) and chest pain (13.1% versus 9.8%, *p* = 0.566) with sequential versus concurrent intrapleural therapy, respectively.

## 4. Discussion

In this retrospective cohort study comparing sequential and concurrent intrapleural therapy for pleural infection, there were no significant differences in treatment failure rate, radiological reduction in effusion size, and adverse events. To date, ours is the largest study comparing concurrent versus sequential intrapleural therapy. An earlier comparative study by Kheir et al. also reported similar outcomes between sequential and concurrent intrapleural therapy, although with a smaller patient cohort with 18 and 20 patients in each arm, respectively [14]. The treatment success rate of concurrent intrapleural therapy observed in our study (94.1%) is comparable with other studies (85–93%) [10–13] that have evaluated concurrent intrapleural therapy.

One concern with concurrent intrapleural therapy is the possibility that concurrent instillation may result in functional changes of DNase (based on manufacturer prescribing information) or the admixed compound, leading to reduced efficacy [17]. However, our reported treatment success rate with concurrent therapy is comparable with established treatment rates observed with sequential intrapleural therapy, suggesting that treatment efficacy is not significantly reduced. In our study, no difference in treatment failure was also observed between sequential and concurrent intrapleural therapy. This is despite a lower number of tPA/DNase doses administered with concurrent intrapleural therapy, where most patients (84.3%) only received one to three doses of tPA/DNase. Considering the high financial cost of alteplase, a hypothetical reduction in drug doses made possible with concurrent therapy may potentially increase the overall cost-effectiveness of treatment. What is more certain are the practical benefits of concurrent intrapleural therapy, which includes a more streamlined protocol that is easier to implement with a shorter treatment time. The duration of clamping of the chest tube is reduced, and there is less frequent access of the chest drainage system which may also potentially reduce the risk for iatrogenic complications.

Of note, pleural bleeding occurred in 9.8% of patients with concurrent intrapleural therapy. This appears to be higher compared to the bleeding rate (4.8%) with sequential intrapleural therapy, although the difference is not statistically significant. A recent study reported a relatively high bleeding rate of 16.1% with concurrent and once-daily intrapleural therapy [13]. However, the reported pleural bleeding rates (2.5–8.9%) from other studies evaluating concurrent intrapleural therapy have otherwise been comparable to expected pleural bleeding rates with sequential intrapleural therapy [18], and there is yet to be a clear signal that concurrent intrapleural therapy is associated with increased bleeding. It is important to keep in mind that interpreting comparisons across studies is not straightforward, due to differences in (1) dose and frequency of intrapleural therapy, (2) clinical risk factors for bleeding, (3) concurrent use of anticoagulation, and (4) definitions of pleural bleeding between studies. In our study, a higher percentage of patients with sequential intrapleural therapy had a deescalated (5 mg of alteplase) dose; whether this reduces the risk of bleeding remains unanswered. However, one study that randomized patients to intrapleural 20 mg alteplase or urokinase reported serious bleeding events in 28% of patients receiving 20 mg alteplase. The bleeding rate decreased to 12% when the dose of alteplase was reduced to 10 mg, suggesting a dose-dependent bleeding risk with intrapleural alteplase [19]. An additional point to consider is demographic variations in patient characteristics such as average body weight may mean that some patients will receive a relatively higher per kg body weight dose of treatment (even with the same dose of 10 mg alteplase administered to all patients).

This also leads to the question of whether pleural infections should be treated with a universal treatment approach (10 mg of alteplase and 5 mg of DNase twice a day), as recommended by a recent expert consensus statement [9]. In our cohort, up to 20% of patients received once-a-day dosing of alteplase, and almost two-thirds (63%) of patients required three or less doses of intrapleural therapy. Increasing evidence supporting the use of once-daily dosing [20] or reduced dosing [12, 21] of intrapleural therapy reinforces the need for further research to guide the treatment of pleural infection. A more personalized approach may plausibly benefit patients with respect to the cost-effectiveness of treatment and minimize the risk of complications such as bleeding.

The main limitations are the retrospective nature of the study and potential confounders due to heterogeneity in the management of pleural infections. Patients with concurrent intrapleural therapy received less doses. Conversely, a higher percentage of patients with sequential intrapleural therapy received a deescalated dose or had intrapleural therapy initiated more than 48 hours following chest drain insertion. Although these variations may complicate the interpretation of our study results, it is also a reflection of the real-world practice of modifying the tPA/DNAse doses based on clinical and radiological response. The lower number of doses of tPA/DNase used with concurrent intrapleural therapy may reflect several responses: (1) increased efficacy with earlier initiation of intrapleural therapy or (2) more effective drainage with concurrent instillation, for which further research is required. Regardless, it is unlikely that a dose modification (5 mg alteplase) would have significantly affected the primary outcome of our study, with a recent study even reporting comparable safety and efficacy with 2.5 mg dosing of alteplase [22]. Lastly, most patients did not have pleural fluid pH tested with a blood gas analyzer. Nevertheless, the positive pleural fluid gram stain or culture of 40% and observed pleural fluid glucose and LDH results were consistent with a patient cohort with pleural infection.

In conclusion, our study adds to the growing literature on the safety and efficacy of concurrent intrapleural therapy in pleural infection and supports this mode of administration as an alternative to sequential intrapleural therapy. In our opinion, with increasing evidence for personalized dosing and frequency [20, 21] of intrapleural alteplase and the potential for complications such as pleural bleeding, there is a need for more research to guide the administration and dosing of intrapleural therapy, particularly in the Asian population where data is lacking.

## Figures and Tables

**Table 1:** Patient demographics and clinical characteristics (*n* = 135).

Age (years)	64 (56-71)
Female gender	30 (22.2)
Ethnicity	
Chinese	96 (71.1)
Malay	22 (16.3)
Indian	14 (10.4)
Charlson's comorbidity score	3 (2-5)
Comorbidities	
Diabetes mellitus	37 (27.4)
Ischemic heart disease or congestive heart failure	21 (15.6)
Ongoing cancer	18 (13.3)
Moderate to severe chronic kidney disease	16 (11.9)
Previous stroke	16 (11.9)
Liver disease	11 (8.1)
Presenting symptoms	
Chest pain	71 (52.6)
Cough	52 (38.5)
Fever	55 (40.7)
Dyspnea	43 (31.9)

Data are presented in number (percentage) and median (interquartile range) for categorical and continuous variables, respectively.

**Table 2:** Clinical characteristics of pleural infection (*n* = 135).

Hospital-acquired infection	19 (14.1)
Right-sided pleural effusion	82 (60.7)
Size of pleural effusion based on radiographic imaging	
Small (<1/3 hemithorax)	11 (8.2)
Moderate (1/3 to 2/3 hemithorax)	62 (45.9)
Large (>2/3 hemithorax)	62 (45.9)
CT or ultrasonographic evidence of pleural loculations	124 (91.9)
Pleural fluid appearance	
Serous or turbid	70 (51.9)
Hemoserous	38 (28.1)
Purulent	27 (20.0)
Gram stain/culture-positive pleural fluid	54 (40.0)
Gram stain/culture-positive pleural fluid or blood	62 (45.9)
Bacteremia	10 (7.4)
Pleural fluid characteristics	
Lactate dehydrogenase (IU/L)	2225 (982-4313)
Glucose (mmol/L)	3.0 (0.6-4.9)
Peripheral leukocyte count (×10^9^/L)	15.2 (10.7-19.6)
C-reactive protein (mg/L)	197 (129-288)
RAPID score	3 (2-4)
Low risk (0-2)	50 (37.0)
Medium risk (3, 4)	56 (41.5)
High risk (5–7)	29 (21.5)

Data are presented in number (percentage) and median (interquartile range) for categorical and continuous variables, respectively. CT: computed tomography.

**Table 3:** Pleural infection bacteriology (*n* = 52).

Culture-positive pleural fluid	52
*Streptococcus viridans*	23 (44.2)
*Streptococcus anginosus* (*anginosus*, *intermedius*, and *constellatus*)	21
*Streptococcus mitis*	2
*Streptococcus salivarius*	1
*Streptococcus pneumoniae*	2 (3.8)
*Staphylococcus aureus*	9 (12.9)
*Klebsiella* spp.	7 (13.5)
*Escherichia coli*	3 (5.7)
*Pseudomonas aeruginosa*	2 (3.8)
Other *Enterobacteriaceae*	5 (9.6)
Anaerobes	10 (19.2)
*Fusobacterium*	5
*Parvimonas micra*	4
Other anaerobes	3
*Mycobacterium tuberculosis*	6 (11.5)
Polymicrobial infection	12 (23.1)

Data are presented in number (percentage). Other *Enterobacteriaceae* are *Proteus mirabilis*, *Enterobacter*, *Citrobacter koseri*, *Morganella morganii*, *Elizabethkingia* spp. Other anaerobes are *Prevotella* spp, *Campylobacter*, and *Pseudopropionibacterium propionicum*.

**Table 4:** Characteristics of chest drainage and intrapleural tPA/DNase therapy (*n* = 135).

Size of chest drain	
8-10 French	61 (45.2)
12-14 French	64 (47.4)
≥16 French	10 (7.4)
Number of chest drains placed	
1 chest drain	107 (79.3)
2-3 chest drains	27 (20.0)
Concurrent antiplatelet or anticoagulation therapy during tPA/DNase therapy	22 (16.3)
Aspirin or clopidogrel	19 (14.1)
Enoxaparin	3 (2.2)
Days from chest drain insertion to initial tPA/DNase therapy	2 (1-4)
First dose of tPA/DNase given within 48 hours of chest drain insertion	79 (58.5)
Number of doses of tPA/DNase therapy administered	
1-2	40 (29.6)
3-4	53 (39.3)
5-6	42 (31.1)
Once-daily dosing of tPA/DNase therapy	28 (20.7)
Deescalated dose of tPA/DNase therapy	15 (11.1)
5 mg alteplase given as first dose	9 (6.7)
5 mg alteplase for all doses	6 (4.4)
Volume of pleural fluid drained (ml)	
Up to 72 hours prior to the first dose of tPA/DNase therapy	167 (79-415)
24 hours after the first dose of tPA/DNase therapy	640 (349-989)
72 hours after the first dose of tPA/DNase therapy	1542 (948-2235)
Decrease in percentage of pleural effusion size (%)^∗^	18.2 (7.5-36.1)
Length of stay from first dose of tPA/DNase (days)	6 (4-11)
Duration of chest drain in pleural cavity following the first dose of tPA/DNase (days)	4 (3-6)

Data are presented in number (percentage) and median (interquartile range) for categorical and continuous variables, respectively. ^∗^Data not available for 8 patients.

**Table 5:** Comparison of patient characteristics, treatment, and outcomes between sequential and concurrent intrapleural therapy.

	Sequential (*n* = 84)	Concurrent (*n* = 51)	*p* value
Age (years)	64 (54-72)	64 (54-72)	0.871
Charlson's comorbidity score	4 (2-6)	4 (2-6)	0.221
Hospital-acquired infection	13 (15.5)	6 (11.8)	0.548
RAPID score	3 (2-5)	3 (2-4)	0.615
Percentage of pleural opacity on CXR before intrapleural therapy^∗^	30.3 (13.4-52.3)	36.5 (26.5-51.4)	0.116
First dose of tPA/DNase given within 48 hours of chest drain insertion	43 (51.2)	36 (70.6)	0.027
Number of doses of tPA/DNase therapy given	4 (3-6)	3 (2-3)	<0.001
≤3 doses of intrapleural therapy	42 (50.0)	43 (84.3)	<0.001
Once-a-day dosing of tPA/DNase therapy	18 (21.4)	10 (19.6)	0.800
5 mg alteplase given as first dose or for all doses	13 (15.5)	2 (3.9)	0.038
Antiplatelet or anticoagulation therapy during tPA/DNase therapy	11 (13.1)	11 (21.6)	0.196
Platelet count (×10^9^/L)	410 (309-527)	388 (313-527)	0.690
Prothrombin time (seconds)	11.3 (10.9-11.9)	11.4 (10.9-11.9)	0.833
Activated partial thromboplastin time (seconds)	31.9 (29.9-34.7)	31.3 (28.5-32.8)	0.109
Pleural fluid drained at 72 hours after intrapleural therapy	1430 (868-2095)	1637 (1135-1637)	0.090
Decrease in percentage of pleural effusion size (%)^‡^	15.1 (6.0-35.7)	26.6 (9.9-38.7)	0.143
Failed therapy or adverse events	22 (26.2)	13 (25.5)	0.928
Failed therapy	8 (9.5)	3 (5.9)	0.534
Required surgery for empyema	2 (2.4)	1 (2.0)	
In-hospital mortality due to pleural infection	5 (6.0)	1 (2.0)	
30-day readmission due to pleural infection	2 (2.4)	1 (2.0)	
Pleural bleeding requiring blood transfusion	4 (4.8)	5 (9.8)	0.298
Pleural bleeding resolved after discontinuation of tPA/DNase therapy	3 (3.6)	4 (7.8)	
Pleural bleeding requiring surgery	1 (1.2)	1 (2.0)	
Chest pain requiring escalation of analgesia	11 (13.1)	5 (9.8)	0.566

Data are presented in number (percentage) and median (interquartile range) for categorical and continuous variables, respectively. CT: computed tomography; CXR: chest X-ray. ^∗^Data missing for 4, 3, and 1 patient/s in the total, sequential, and concurrent therapy groups, respectively. ^‡^Data missing for 8, 7, and 1 patient/s in the total, sequential, and concurrent therapy groups, respectively.

## Data Availability

The data that support the findings of this study are available from the corresponding author upon reasonable request.
